# The Perceptions and Experiences of Medical Faculty on the Undergraduate Mentoring System

**DOI:** 10.7759/cureus.72242

**Published:** 2024-10-23

**Authors:** Padmakumar Krishnankutty Nair

**Affiliations:** 1 Forensic Medicine, Jubilee Mission Medical College and Research Institute, Thrissur, IND

**Keywords:** medical faculty, medical students, mentoring program, mentoring system in medical education, mentorship, perception

## Abstract

Background and aims

Mentoring programs for medical students in medical institutions are essential for the benefit of both mentors and mentees. The present study aimed to explore the perceptions and experiences of medical faculty regarding the effectiveness and challenges of the undergraduate mentoring system in medical education.

Materials and methods

A descriptive cross-sectional questionnaire-based study was conducted among 122 medical faculty members. Data were collected using a structured questionnaire consisting of 20 questions via Google Forms (Google, Mountain View, California) through e-mail and WhatsApp. The collected data were expressed as frequencies and proportions.

Results

Among 122 respondents, the percentage of female and male faculty members were 54.1% and 45.9%, respectively. The majority of respondents (78.7%) do not receive formal training for mentoring, highlighting a potential area for improvement in the program. Most respondents (67.2%) felt comfortable or very comfortable addressing personal issues, indicating a positive environment for personal discussions. Of the medical faculty, 77.9% felt that the mentorship program contributes to personal development, while 84.4% of medical faculty members believe the program helps academic development. Institutional support for the mentoring program has been perceived as good by 68.9% of medical faculty members.

Conclusion

This study concludes that enhancing the undergraduate mentoring system in medical education necessitates a collaborative effort from faculty, administration, and students. By acknowledging and addressing the challenges identified in this study, medical colleges can create a more robust mentoring framework that not only supports faculty development but also enriches the educational journey of medical students.

## Introduction

Mentorship is defined as "a process whereby an experienced, highly regarded, an empathic person (the mentor) guides another individual (the mentee) in the development and examination of their ideas, learning, and personal and professional development" by the Standing Committee on Postgraduate Medical and Dental Education (SCOPME) [1]. There has been a lot of research on the advantages of mentorship in health professions education [2]. Focused and strong mentorship has been linked to enhanced mentee productivity, self-efficacy, career satisfaction, and a sense of belonging and support [3]. Learners who participate in active mentorship relationships are more likely to persist in their academics and make positive academic and social decisions [4,5], with positive mentoring being cited as the most important factor in the completion of studies [6].

Beyond teaching, a mentor must perform a variety of functions in medical education, such as supervisor, counselor, and many more, and in order to carry out these diverse responsibilities, building relationships is unquestionably necessary [7]. Mentorship programs are increasingly common in undergraduate medical, dental, and nursing colleges in India, and many positive effects have been reported. Most studies on mentorship in Indian medical colleges have predominantly focused on the mentee's perspective. These studies typically explore the strengths and weaknesses of programs [8-11].

Some studies have recommended the implementation of formal mentoring programs for medical students in medical institutions [7,12], while some studies have reviewed the benefits of these programs for both mentees and mentors [13-15].

While the mentee's perspective has been well-documented, there is a significant gap in research regarding the mentor's perspective. Faculty knowledge, attitudes, and practices regarding mentorship have not been deeply explored. There is a dearth of research on mentoring in medical education in the Indian context. Such a study will provide a comprehensive understanding of the mentorship process and will lead to more effective and sustainable mentorship programs in medical colleges.

Aim of the study

To find out the personal experiences of medical faculty members in mentoring undergraduates, including positive outcomes and challenges faced. The study also seeks to understand faculty members' views on the overall effectiveness of the mentoring system, its impact on undergraduate education, and areas for potential improvement.

Research question

What are the perceptions and experiences of medical faculty regarding the effectiveness and challenges of the undergraduate mentoring system in medical education?

## Materials and methods

Ethical approval

Ethical approval was obtained from the institutional ethical committee before the commencement of the study with reference number (109/24/IEC/JMMC&RI). 

Participants and sample size

A descriptive cross-sectional questionnaire-based study was conducted among 122 medical faculty members from various medical colleges in Kerala, India, in August 2024. Samples were collected using the non-probability (convenience) sampling method. Among 2400 medical faculty members from 18 medical colleges of Kerala, 122 medical faculty members were selected conveniently on the basis of experience in the mentorship program, and the students were assigned randomly to the mentees.

Inclusion criteria for this study were medical faculty who are working in various medical colleges in Kerala and who is/was a mentor for undergraduate students. Medical faculty members who had no mentoring experience were excluded from this study.

Calculation of sample size

Based on the proportion of perception of participants (mentors) observed in an earlier publication [16] with 95% confidence level and 15% relative allowable error, the minimum sample size comes to 102.

n=((Z₁ - )^2^×p × q)/d^2^, where Z₁ - α⁄2 = 1.96, p = 0.625, q = 0.375, and d = 15% of p.

Finally, the author included 122 medical faculty members for the conduction of the current study.

Data collection instruments

A pretested, structured questionnaire was used as a study tool. The questionnaire consisted of five sections. Section A, with four items, was designed to collect demographic characteristics such as gender, age, years of experience, and current designation. Section B contained four questions about the concept and practice of the mentorship program; section C included eight questions items about the perception of faculties on the mentorship program; section D was about challenges faced by faculties during the mentorship program with one open-ended question; and section E contained three items about suggestions and feedbacks on mentorship program.

The questionnaire consisted of 20 questions, including both open-ended and closed-ended questions.

Pilot testing

A questionnaire was designed based on the meticulous and extensive literature review, opinions of medical experts, and the mentorship experiences of medical faculties. Additionally, it was validated by four experienced medical professors and experts in medical education and mentoring. The self-designed questionnaire was pilot-tested and validated to ensure the face and content validity. The pilot testing was carried out in the month of August 2024 among 20 experienced medical faculties, and face validity was verified. Non-probability sampling was used to select participants randomly. These participants were excluded from the main study. The pilot testing was done to evaluate the feasibility of the survey and to finalize the questionnaire. Based on the pilot testing, the questionnaire was appropriately revised to make the questionnaire more standard.

Data collection

Data were collected using a structured questionnaire via Google Forms through e-mail and WhatsApp. Informed consent was obtained from all participants before the commencement of the study. All the participants were provided with clear, comprehensive information about the study purpose, what participation involved, potential risks and benefits, and their rights as participants. The rights of participants, which include privacy, confidentiality, and anonymity, were ensured in the present study. Some of the incomplete responses were discarded, and finally, 122 respondents' questionnaires were collected for the analysis.

Data analysis

The data were recorded in an Excel sheet (Microsoft, Redmond, Washington), and descriptive analysis was done using SPSS version 20.0 (IBM Inc., Armonk, New York). The data are expressed as frequencies and proportions.

## Results

Table 1 presents a demographic characteristic of a sample of 122 respondents.

**Table 1:** Demographic characteristics

Variables	Frequency	Percentage
Gender		
Male	56	45.9
Female	66	54.1
Age (in years)		
Below 30	1	0.8
31-40	62	50.8
41-50	26	21.3
51-60	26	21.3
61-70	7	5.7
Years of experience		
Less than 2 years	15	12.3
2 to 5 years	45	36.9
5 to 10 years	28	23
More than 10 years	34	27.9
Current position		
Assistant professor	51	41.8
Associate professor	22	18
Professor	49	40.2
Total	122	100

The gender distribution of the respondents indicates a slight female majority, with women making up 54.1% of the sample compared to 45.9% of male respondents. This suggests a fairly balanced representation, although there is a noticeable leaning towards female participants.

The age distribution shows that the majority of respondents (50.8%) are aged between 31 and 40 years, indicating that this age group is the most represented in the sample. There is a small proportion (0.8%) of respondents younger than 30, while the age groups 41-50 and 51-60 each represent an equal share of 21.3%. A smaller segment (5.7%) comprises those aged 61-70. This distribution reveals that the workforce is predominantly comprised of individuals in their 30s, with fewer respondents in older age brackets.

In terms of professional experience, 36.9% of respondents have between two and five years of experience, which is the largest group within this variable. Those with less than two years of experience comprise a smaller segment (12.3%). The respondents with 5 to 10 years of experience account for 23%, while those with more than 10 years of experience make up 27.9%. This indicates a rather diverse level of experience among the respondents, with a significant proportion being relatively early in their career (less than five years of experience) but also a noteworthy number having substantial experience (10+ years).

Table 2 categorizes the respondents according to their current academic positions. The largest group consists of Assistant Professors, representing 41.8% of the total sample, closely followed by Professors at 40.2%. Associate Professors form the smallest group, comprising 18%. This distribution implies a well-balanced representation of academic ranks, with a predominance of early-career and mid-career academics.

**Table 2:** Concept and practice of mentorship program

Response of questions	Frequency	Percentage
How would you rate the overall structure of the current mentoring program?		
Poor	9	7.4
Average	48	39.3
Good	60	49.2
Excellent	5	4.1
How often do you meet with our mentees?		
Weekly	12	9.8
Bi-weekly	9	7.4
Monthly	50	41
Quarterly	9	7.4
As needed	42	34.4
How are mentees assigned to you?		
Randomly	95	77.9
Based on specialty	9	7.4
Based on mentee’' choice	6	4.9
Others	12	9.8
Do you receive any formal training for mentoring?		
Yes	26	21.3
No	95	78.7
Total	122	100

Table 2 presents the results of a questionnaire designed to evaluate various aspects of a mentoring program. Regarding the overall structure of the mentoring program, the majority of respondents (49.2%) rated the program as "good", followed by 39.3% as "average", and 7.4% as "poor". Only 4.1% of faculty members rated this mentorship program as "excellent".

Regarding the frequency of meetings with mentees, the majority of respondents (41% + 34.4% = 75.4%) either meet with their mentees monthly or as needed, suggesting a flexible mentoring schedule.

The majority (77.9%) of mentees are assigned randomly, indicating a standardized approach to assigning mentoring pairs in this program. The majority of respondents (78.7%) do not receive formal training for mentoring, highlighting a potential area for improvement in the program.

**Table 3:** Perception of faculties on mentorship program

Response of questions	Frequency	Percentage
How would you describe our relationship with our mentees?		
Very strong	2	1.6
Strong	42	34.4
Neutral	62	50.8
Weak	12	9.8
Very weak	4	3.3
How comfortable do you feel in addressing personal issues with our mentees?		
Very comfortable	9	7.4
Comfortable	73	59.8
Neutral	36	29.5
Uncomfortable	3	2.5
Very uncomfortable	1	0.8
How comfortable do you feel in addressing academic issues with our mentees?		
Very comfortable	42	34.4
Comfortable	69	56.6
Neutral	10	8.2
Uncomfortable	1	0.8
Very uncomfortable	0.0	0.0
How do you typically communicate with our mentees?		
In-person meetings	50	41
In-person meetings, messaging apps (e.g., WhatsApp)	28	23
In-person meetings, phone calls, messaging apps (e.g., WhatsApp)	25	20.5
In-person meetings, phone calls	6	4.9
Phone calls, messaging apps (e.g., WhatsApp)	4	3.3
How effective do you believe the mentoring program is in achieving its goals?		
Very effective	14	11.5
Very effective	60	49.2
Neutral	39	32
Ineffective	9	7.4
To what extent do you think the mentoring program helps mentees in their personal development?		
A great extent	25	20.5
A moderate extent	70	57.4
A slight extent	26	21.3
Not at all	1	0.8
To what extent do you think the mentoring program helps mentees in their academic development?		
A great extent	31	25.4
A moderate extent	72	59
A slight extent	19	15.6
A slight extent	0.0	0.0
How would you rate the support provided by the institution for the mentoring program?		
Excellent	20	16.4
Good	64	52.5
Average	28	23
Poor	10	8.2
Total	122	100

Table 3 presents details of the perceptions and experiences of mentors with their mentees regarding mentorship programs in a medical college. Regarding relationships with mentees, the majority of respondents (85.2%) reported at least a strong or neutral relationship (34.4% strong, 50.8% neutral), but a small percentage (13.1%) indicated a weak or very weak relationship.

Most respondents (67.2%) felt comfortable or very comfortable addressing personal issues, indicating a positive environment for personal discussions. A high percentage (91%) of mentors felt at least comfortable discussing academic issues, showing a strong willingness to engage in academic mentoring.

The majority (41%) use in-person meetings primarily for communication, suggesting a preference for direct interaction over digital methods. Twenty-three percent of faculty members used a combination of in-person meetings and messaging apps (e.g., WhatsApp), while 20.5% used in-person meetings, phone calls, messaging apps (e.g., WhatsApp), followed by combined use of in-person meetings and phone calls(4.9%) and phone calls and messaging apps (e.g., WhatsApp) by only 3.3%.

Regarding the effectiveness of the mentoring program, a combined 60.7% rated the program as very effective (11.5%) or effective (49.2%), indicating satisfaction with the program's outcomes among mentors, while 32% were neutral, and only 7.4% judged the mentorship program ineffective.

Regarding the mentoring program's impact on personal development, a significant majority (77.9%) of medical faculty felt that the program contributes to personal development to a great (20.5%) or moderate extent (57.4%). Of the medical faculty, 21.3% said the mentoring program's impact was a slight extent, and negligible (0.8%) responded not at all.

Similar to personal development, 84.4% of medical faculty members believe the program helps academic development to a great (25.4%) or moderate extent (59%), followed by 15.6% of a slight extent.

Institutional support for the mentoring program has been perceived as good by the medical faculty members. About 68.9% rated institutional support as good (52.5%) or excellent (16.4%), indicating a generally positive view of the support received. Twenty-three percent of doctors rated the mentorship program as average regarding institutional support, while only 8.2% regarded it as poor.

Table 4 outlines various challenges faced by mentors or advisors, categorized by response type, along with their corresponding frequencies and percentages. Lack of time is the most frequently cited barrier, with 22 responses (18%), followed by the combination of lack of time and lack of training (15.6%), while lack of training alone is reported by 14 respondents (11.5%).

**Table 4:** Challenges faced by faculties during the mentorship program

Response of questions	Frequency	Percentage
Lack of time	22	18
Lack of time, lack of training	19	15.6
Lack of training	14	11.5
Lack of training, difficulty in building relationships	10	8.2
Lack of time, difficulty in building relationships	8	6.6
Difficulty in building relationships	7	5.7
Lack of time, lack of training, difficulty in building relationships	7	5.7
Difficulty in building relationships, mismatched expectations	6	4.9
Mismatched expectations	5	4.1
Lack of time, lack of training, difficulty in building relationships, mismatched expectations	4	3.3
Lack of time, mismatched expectations	3	2.5
Lack of time, difficulty in building relationships, mismatched expectations	2	1.6
Lack of time, lack of training, mismatched expectations	2	1.6
Lack of time, lack of training, mismatched expectations, not in sync with present generation's mentality	2	1.6
Lack of training, mismatched expectations	2	1.6
Lack of time, Lack of time for mentee and mentor	1	0.8
Lack of time, lack of training, cooperation of mentee	1	0.8
Lack of time, lack of training, difficult to draw boundaries. Now keeping at a distance. More at a formal side	1	0.8
Lack of time, lack of training, difficulty in building relationships, mismatched expectations, students are not open to faculty. They are hesitant to meet their mentors. Their attitude shows to leave them alone.	1	0.8
Lack of time, lack of training, workload	1	0.8
Lack of time, mismatched expectations, Some students do not come at all, some come. They have their own way of solving problem	1	0.8
Lack of time, students are burdened with too much work so its very difficult to get time to meet them	1	0.8
Lack of training, difficulty in building relationships, mismatched expectations	1	0.8
Students are not interested in this program. They come once or twice then they won’t come	1	0.8
Total	122	100

The response indicating a combination of lack of training and difficulty in building relationships had 10 responses (8.2%). Similar challenges with both lack of time and difficulty in building relationships are reflected, with eight responses (6.6%). Difficulty in building relationships by itself was mentioned by seven respondents (5.7%). One of the more complex issues is the combination of lack of time, lack of training, and difficulty in building relationships, which also received seven responses (5.7%).

Issues of mismatched expectations appear as a barrier in various combinations: alone was noted in five responses (4.1%), alongside other factors such as difficulty in building a relationship was noted by six respondents (4.9%).

The most complex combination of all four factors; lack of time, lack of training, difficulty in building relationships, and mismatched expectations, was reported by four respondents (3.3%).

Several responses (only 1) highlight unique challenges, including lack of time related to lack of time specifically for both mentee and mentor.

Table 5 illustrates responses regarding the desired improvements for a mentorship program, accompanied by the frequency of each response and the corresponding percentage of total responses.

**Table 5:** Suggestions by faculties on the mentorship program

Response of questions	Frequency	Percentage
More structured training for mentors	16	13.1
More structured training for mentors, more institutional support, clearer guidelines and objectives	13	10.7
More structured training for mentors, clearer guidelines and objectives	9	7.4
More structured training for mentors, better matching process for mentors and mentees, clearer guidelines and objectives	8	6.6
More structured training for mentors, better matching process for mentors and mentees, increased frequency of meetings	7	5.7
More structured training for mentors, better matching process for mentors and mentees, increased frequency of meetings	7	5.7
Better matching process for mentors and mentees, Increased frequency of meetings	6	4.9
More structured training for mentors, better matching process for mentors and mentees, more institutional support, clearer guidelines and objectives	5	4.1
More structured training for mentors, increased frequency of meetings, more institutional support, clearer guidelines and objectives	5	4.1
More structured training for mentors, increased frequency of meetings	5	4.1
More structured training for mentors, more institutional support	5	4.1
More institutional support	4	3.3
More structured training for mentors, better matching process for mentors and mentees	4	3.3
More structured training for mentors, increased frequency of meetings, clearer guidelines and objectives	4	3.3
Better matching process for mentors and mentees	3	2.5
Clearer guidelines and objectives	3	2.5
Increased frequency of meetings	3	2.5
More structured training for mentors, increased frequency of meetings, more institutional support	2	1.6
More structured training for mentors, better matching process for mentors and mentees, more institutional support	2	1.6
More structured training for mentors, better matching process for mentors and mentees, increased frequency of meetings	2	1.6
Increased frequency of meetings, more institutional support	2	1.6
Better matching process for mentors and mentees, Increased frequency of meetings, more institutional support, peer mentor	1	0.8
Increased frequency of meetings, clearer guidelines and objectives	1	0.8
Increased frequency of meetings, more institutional support, clearer guidelines and objectives	1	0.8
More structured training for mentors, better matching process for mentors and mentees, clearer guidelines and objectives	1	0.8
More structured training for mentors, better matching process for mentors and mentees, increased frequency of meetings	1	0.8
More structured training for mentors, Better matching process for mentors and mentees, increased frequency of meetings	1	0.8
More structured training for mentors, increased frequency of meetings, more institutional support, clearer guidelines and objectives	1	0.8
Total	122	100

The most frequently suggested improvement is "more structured training for mentors," noted by 16 respondents (13.1% of total responses). This indicates a strong demand for enhancing the training framework for mentors.

Responses that incorporate multiple suggestions are prevalent. For example, the combination of "more structured training for mentors," "more institutional support," and "clearer guidelines and objectives" received 13 votes (10.7%). Several suggestions highlight the interactions between training, institutional support, clear guidelines, and mentor-mentee matching processes, indicating interconnectedness in what participants believe will improve the mentorship experience. "More structured training for mentors" was a recurring theme in many responses, appearing in the majority of the combinations listed, highlighting its importance across different contexts.

Other notable suggestions include: "Better matching process for mentors and mentees" (noted in several responses). "Increased frequency of meetings" illustrates the interest in promoting more engagement between mentors and mentees.

The least frequent suggestions (one response each, accounting for 0.8% of the total) indicate a desire for very specific combinations of improvements, such as peer mentoring programs and improved guidelines.

Table 6 describes responses regarding the consideration of feedback from mentors in improving the mentoring program. The majority of respondents (53.3%) believe that mentor feedback is adequately taken into account in program improvements. A significant portion of respondents (32.8%) are ambiguous about the acknowledgment of mentor feedback, indicating uncertainty about the program's responsiveness. A smaller group of respondents (13.9%) feel that their feedback is not sufficiently considered, suggesting some degree of dissatisfaction.

**Table 6:** Feedbacks by faculties on the mentorship program

Response of questions	Frequency	Percentage
Do you feel that the feedback from mentors is adequately considered in improving the mentoring program?		
No	17	13.9
Unsure	40	32.8
Yes	65	53.3
Total	122	100

## Discussion

The purpose of this study was to explore the perceptions and experiences of medical faculty regarding the effectiveness and challenges of the undergraduate mentoring system in medical education by analyzing the questionnaire collected from 122 respondents.

The age distribution of the current study shows that the majority of respondents (50.8%) are aged between 31 and 40 years, indicating that this age group is the most represented in the sample.

The findings of the present study reflect a favorable perception of the mentoring program among participants, with strong relationships, comfort in discussing issues, effective communication methods, perceived effectiveness, and strong institutional support. The responses indicate that mentors generally feel equipped to engage with their mentees, both personally and academically, and believe in the program's positive impact on mentees' development.

The findings of our study on the mentoring program align well with previous research on mentoring relationships, particularly in academic and medical settings.

Our study indicates a favorable perception of the mentoring program among participants, noting strong relationships and comfort in discussing issues. This mirrors findings from a qualitative study at Semmelweis University, where mentors expressed satisfaction with their mentoring program, emphasizing the effectiveness of communication and the benefits of supervision sessions [17]. Similarly, other studies have highlighted the importance of open communication, mutual respect, and trust as key components of effective mentoring relationships [18, 13].

Participants in our study felt that mentors were well-equipped to engage with mentees, both personally and academically. This is consistent with previous research that found mentors reported significant professional growth through their roles, including enhanced communication and problem-solving skills, which are crucial in medical training [17, 19]. The ability of mentors to provide effective guidance and support is crucial, as noted in studies where mentors gained insights and developed essential skills that benefited both themselves and their mentees [13].

The strong institutional support noted in our study is echoed in other research, which emphasizes that successful mentoring programs often rely on robust institutional backing. This support can lead to improved outcomes for both mentors and mentees, fostering a culture of engagement and satisfaction within academic environments [13,20]. For instance, studies have shown that institutional support contributes to higher retention rates and overall job satisfaction among faculty involved in mentoring programs [13].

Our findings regarding the perceived positive impact of the mentoring program on mentees' development are also supported by previous studies. Research indicates that mentees benefit from improved confidence, skill development, and a sense of community, which are essential for their professional growth [19]. This aligns with our observations that mentees experience personal and academic growth through the mentoring relationship.

Our study on the mentoring program highlights several key aspects that resonate with findings from previous research in similar settings. The current study indicates that the mentoring program is generally viewed positively regarding its structure. This is consistent with other studies, such as the one conducted at a different Indian medical college, which found that students recognized the importance of mentoring for personal and professional development. These studies emphasize that structured mentoring programs can foster a supportive environment for students, enhancing their academic and emotional well-being [21, 22].

The perceived lack of formal training for mentors (78.7%) in our study aligns with findings from other research, which suggests that many mentors in Indian medical institutions feel unprepared for their roles. For instance, a study highlighted the need for structured training workshops to equip mentors with necessary skills, such as building trust and effective communication. This lack of preparation can hinder the effectiveness of mentoring relationships, as mentors may struggle to provide the guidance that mentees need [21, 23].

Our observation about the flexibility of meeting frequencies is echoed in other studies, where flexibility is seen as beneficial for accommodating the varying schedules of both mentors and mentees. However, while flexibility is important, some research suggests that regular, scheduled meetings can enhance the mentoring experience by establishing consistency and accountability in the mentor-mentee relationship [22].

The random assignment of mentees in our study, while promoting diversity, raises concerns about the potential mismatch between mentors and mentees. Previous studies have noted that personalized matching based on specialties or interests can significantly improve the mentoring experience. For example, a study indicated that when mentees are paired with mentors who share similar academic interests or career goals, the mentoring relationship tends to be more productive and fulfilling [23, 7].

The suggestion for more personalized matching in our findings is supported by literature that emphasizes the importance of tailoring mentoring relationships to meet individual needs. Research has shown that personalized mentorship can lead to better outcomes in terms of mentee satisfaction and professional development, highlighting the necessity for institutions to consider mentee preferences during the assignment process [22].

Our study highlights that the most significant barriers to effective mentoring among medical faculty members are lack of time and lack of training, along with difficulties in building relationships and mismatched expectations. These barriers resonate with previous research that has explored similar challenges in mentoring programs within medical education.

Studies [16, 24] found that mentors reported lack of time as a major barrier to successful mentoring relationships, emphasizing that competing responsibilities severely limit their availability for mentoring activities. Similarly, another study noted that faculty members often face overwhelming demands from clinical and administrative duties, which detracts from their ability to engage in mentorship effectively [25]. This aligns with our findings, where lack of time emerged as a primary obstacle.

The lack of training for mentors is another barrier that has been widely documented. Research indicates that many faculty members feel unprepared for the mentoring role due to insufficient training in mentorship skills [26]. This lack of preparation can lead to ineffective mentorship experiences, as mentors may struggle to provide the necessary guidance and support to their mentees. Our results corroborate this issue, highlighting the need for structured training programs to equip mentors with the skills needed to foster effective mentoring relationships.

Building strong mentor-mentee relationships is crucial for effective mentoring, yet many studies have identified challenges in this area. A study focusing on pediatric hospital medicine revealed that mismatched expectations and a lack of available mentors significantly hinder relationship development [25]. This aligns with our findings, which also identified difficulties in establishing and maintaining effective relationships as a barrier to mentoring success.

Mismatched expectations between mentors and mentees can lead to frustration and disengagement, a challenge noted in various studies. For example, participants in one study expressed that unclear boundaries and differing goals often strained mentoring relationships [25]. This theme is echoed in our findings, where mismatched expectations were highlighted as a significant barrier, indicating the need for clear communication and alignment of goals at the outset of mentoring relationships.

Overall, the barriers identified in our study, such as lack of time, lack of training, difficulties in building relationships, and mismatched expectations, are well-documented in the literature on mentoring programs for medical faculty. Addressing these challenges through institutional support, training programs, and clear communication strategies can enhance the effectiveness of mentoring relationships and ultimately contribute to the professional development of medical faculty members.

The present study's findings highlight several critical gaps in the field of medical education mentoring. Addressing these gaps, such as optimizing mentoring time, enhancing mentor training, understanding interpersonal dynamics, managing expectations, and implementing robust support structures, could significantly improve the effectiveness of mentoring programs. Future research should focus on these areas to develop evidence-based practices that enhance the mentoring experience for both mentors and mentees in medical education.

The study is highly relevant in the current context, as mentoring programs are gaining increasing importance in medical education in India. By identifying key barriers, the findings can inform strategies to improve the effectiveness of mentoring initiatives. The study explores a range of barriers, including lack of time, lack of training, difficulties in building relationships, and mismatched expectations. This holistic approach provides a nuanced understanding of the challenges faced in mentoring programs. The study's findings resonate with existing literature on mentoring in medical education, both in India and globally. This alignment strengthens the validity of the results and suggests that the identified barriers are common across different contexts.

The study's findings have clear practical implications for improving mentoring programs in Indian medical colleges. The barriers identified can serve as a starting point for institutions to develop targeted interventions and support mechanisms for mentors and mentees.

Strength and limitations

The present study's strengths lie in its timeliness, comprehensive exploration of barriers, alignment with existing literature, emphasis on interconnectedness, identification of the need for enhanced support, potential for practical applications, and contribution to an underexplored area of research in Indian medical education.

The present study provides valuable insights into the perceptions and experiences of medical faculty on the undergraduate mentoring system; it also has some limitations that should be acknowledged. The study's findings are context-specific to some medical colleges in India, which may limit the generalizability of the results to other institutions or regions. Different medical colleges may have varying structures, cultures, and resources that influence mentoring programs, making it difficult to apply these findings universally.

The study primarily relies on self-reported data from participants, which can introduce biases such as social desirability bias or respondent bias. Participants may provide responses that they believe are more favorable or acceptable rather than their true experiences and perceptions. This can affect the accuracy of the data collected. A larger and more diverse sample could provide a more comprehensive understanding of the barriers faced by both mentors and mentees.

Future research could address these limitations to provide a more comprehensive understanding of mentoring in medical education.

## Conclusions

This study has provided valuable insights into the perceptions and experiences of medical faculty regarding the effectiveness and challenges of the undergraduate mentoring system in medical education. The findings highlight the critical role that mentoring plays in shaping the academic and professional development of medical students, emphasizing its potential to enhance learning outcomes, emotional well-being, and career trajectories.

The study also reveals significant barriers that impede the effectiveness of mentoring programs. The lack of time, insufficient training for mentors, difficulties in building relationships, and mismatched expectations between mentors and mentees are prevalent challenges that require urgent attention. These barriers not only hinder the development of strong mentor-mentee relationships but also diminish the overall impact of mentoring on students' educational experiences. To address these challenges, it is essential for medical institutions to implement structured training programs for mentors, allocate dedicated time for mentoring activities, and establish clear expectations for both mentors and mentees. 
